# A Phase 1 Open-Label Study to Assess the Tolerability, Safety, and Immunogenicity of Hyaluronidase-Facilitated Subcutaneous Immunoglobulin 20% in Healthy Adults

**DOI:** 10.1007/s10875-023-01632-2

**Published:** 2023-12-22

**Authors:** Andras Nagy, Kimberly Duff, Alexander Bauer, Fred Okonneh, Juan Carlos Rondon, Leman Yel, Zhaoyang Li

**Affiliations:** 1 Baxalta Innovations GmbH, a Takeda Company, Vienna, Austria; 2 Takeda Development Center Americas, Inc., Cambridge, MA USA; 3 Clinical Pharmacology of Miami, LLC, an Evolution Research Group portfolio company, Miami, FL USA; 4 Present Address: University of California, Irvine, CA USA

**Keywords:** Hyaluronidase-facilitated subcutaneous immunoglobulin 20%, inborn errors of immunity, primary immunodeficiency disease, recombinant human hyaluronidase, safety, tolerability

## Abstract

**Purpose:**

Hyaluronidase-facilitated subcutaneous immunoglobulin (fSCIG) 20% will allow reduced infusion volumes and frequency versus existing subcutaneous therapies such as fSCIG 10% and conventional subcutaneous immunoglobulin 20%, respectively. We assessed the tolerability, safety, and immunogenicity of warmed and unwarmed fSCIG 20%.

**Methods:**

This phase 1, single-dose, open-label, three-arm study enrolled healthy adults aged 19–50 years (inclusive) at a single US center (NCT05059977). Post-screening, participants received a single fSCIG 20% dose comprising recombinant human hyaluronidase and varying doses of in-line warmed or unwarmed immunoglobulin G (IgG) during a 4-day treatment period in a sentinel and sequential dosing design (treatment arm 1, warmed IgG 20% 0.4 g/kg; treatment arm 2, warmed IgG 20% 1.0 g/kg; treatment arm 3, unwarmed IgG 20% 1.0 g/kg). Participants were followed for 12 (± 1) weeks post-infusion. The primary endpoint was tolerability (“tolerable” infusions were not interrupted, stopped, or reduced in rate owing to fSCIG 20%-related treatment-emergent adverse events (TEAEs)). Secondary endpoints included occurrence of TEAEs.

**Results:**

Overall, 24 participants were included, 8 per treatment arm (mean age 39.0 years, 54.2% men). All participants tolerated the infusions. All TEAEs were mild (107 events, in all participants), and all participants experienced fSCIG 20%-related (105 events) and local (102 events) TEAEs. Infusion site erythema and infusion site swelling were most frequently reported. No serious TEAEs occurred, and no participants discontinued the study owing to TEAEs.

**Conclusion:**

fSCIG 20% was well-tolerated with a favorable safety profile in healthy adults. Future studies will evaluate fSCIG 20% in primary immunodeficiency diseases.

**Trial registration number (ClinicalTrials.gov):** NCT05059977 (registered 28 September 2021).

**Supplementary Information:**

The online version contains supplementary material available at 10.1007/s10875-023-01632-2.

## Introduction

Immunoglobulin therapies are used to treat various medical conditions, including primary immunodeficiency diseases (PIDs) and autoimmune neuromuscular diseases such as chronic inflammatory demyelinating polyradiculoneuropathy or multifocal motor neuropathy [1, 2]. PIDs, also referred to as inborn errors of immunity, encompass a diverse group of conditions that affect the functioning of the immune system, with antibody deficiencies representing the most common type of PIDs [3–5]. PIDs may be treated using immunoglobulin replacement therapy (IgRT), which includes subcutaneous immunoglobulin (SCIG) and intravenous immunoglobulin (IVIG) treatments, and is used to reduce risk of infection in patients with PIDs caused by impairments in antibody production [1–3, 6].

Compared with IVIG, SCIG is associated with fewer systemic adverse reactions and can be self-administered at home instead of at a clinic or hospital by a healthcare professional [1, 7, 8]. However, the volume of conventional SCIG treatment that can be infused subcutaneously is limited, which results in the need for more frequent infusions with SCIG than with IVIG [1, 7, 8]. The use of conventional SCIG also necessitates multiple infusion sites, which may deter some patients from receiving SCIG therapy. As an example, patient preference studies of IgRT in PIDs have demonstrated patient and caregiver preference for fewer needle sticks per treatment [1, 9, 10].

Various strategies have been adopted to mitigate the limitations associated with conventional SCIG treatment. Hyaluronidase-facilitated subcutaneous immunoglobulin 10% (fSCIG 10%; HyQvia/HYQVIA; Baxalta US, Inc., a member of the Takeda group of companies, Lexington, MA, USA) is an infusion of human immunoglobulin G (IgG) 10% and recombinant human hyaluronidase (rHuPH20) [11, 12]. rHuPH20 acts to depolymerize hyaluronan in the extracellular matrix, which transiently increases the permeability of subcutaneous tissue, allowing larger volumes of IgG (up to 600 mL) to be administered at a single infusion site [11–13]. Conventional SCIG 20% therapies are concentrated formulations that permit infusion of the same dose in smaller volumes than less-concentrated (e.g., 10% IgG) SCIG treatments [1, 2, 14–18].

Given that many patients with PIDs require lifelong treatment, the development of new therapies that allow for greater treatment individualization and increased convenience is anticipated to offer meaningful patient benefit (such as through potentially decreased infusion times) and improve the overall patient treatment experience [13, 19]. Hyaluronidase-facilitated subcutaneous immunoglobulin 20% (fSCIG 20%, also known as TAK-881) is an infusion of IgG 20% and rHuPH20. The ratio of IgG to rHuPH20 is the same as for fSCIG 10%. By combining the potential benefits of fSCIG 10% and conventional SCIG 20%, fSCIG 20% will allow for smaller infusion volumes than fSCIG 10% and less frequent infusions than conventional SCIG 20% therapy to achieve the same target monthly immunoglobulin dose [20]. fSCIG 20% has previously been evaluated in a preclinical study that confirmed the feasibility of administering in-line warmed fSCIG 20% to pigs at infusion rates of up to 450 mL/h [20]. Higher IgG concentrations have been associated with increased viscosity compared with less-concentrated IgG therapies, resulting in increased in-line pressure, reduced infusion rates, and longer infusion times per month [20]. Given that warming IgG to 37 °C reduced its viscosity in the preclinical study of fSCIG 20% [20], assessing the effect of in-line warming on IgG viscosity in humans versus unwarmed conditions would improve understanding of optimal infusion parameters.

This phase 1, open-label study investigated the tolerability, safety, and immunogenicity of warmed and unwarmed fSCIG 20% at various infusion rates in healthy adults. The primary objective was to assess the tolerability of fSCIG 20%; assessing the safety and immunogenicity of fSCIG 20% were secondary objectives.

## Methods

### Study Design

**Fig. 1:**
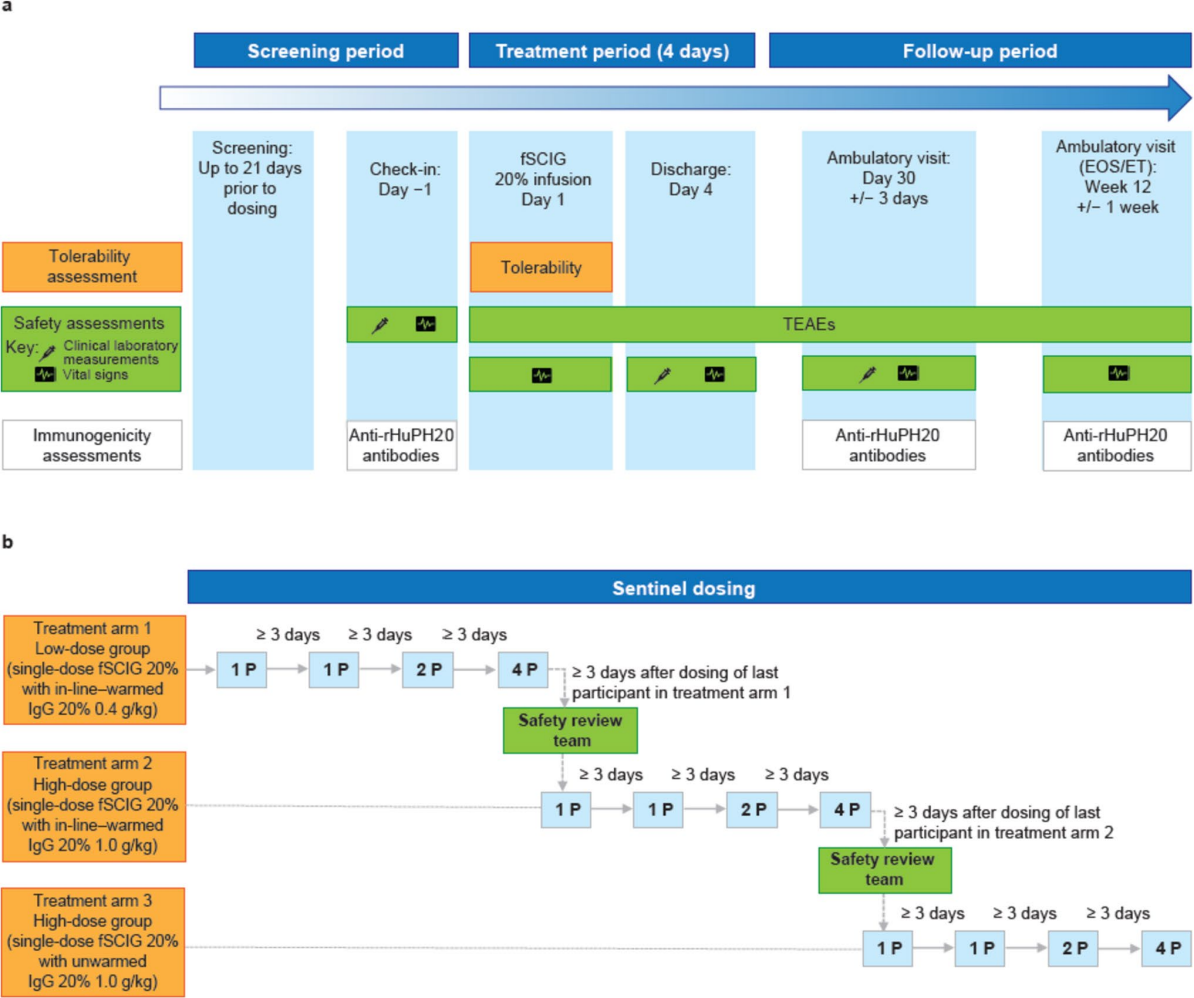
Overall study design **a** and sentinel dosing design **b**. Abbreviations: *EOS*, end of study; *ET*, early termination; *fSCIG 20%*, hyaluronidase-facilitated immunoglobulin 20%; *IgG*, immunoglobulin G; *P*, participant; *rHuPH20*, recombinant human hyaluronidase; *TEAE*, treatment-emergent adverse event

This phase 1, single-dose, open-label, three-arm study was conducted at a single center in the USA between October 12, 2021, and April 12, 2022 (NCT05059977). The study consisted of a screening period of up to 21 days before fSCIG 20% dosing, a 4-day study treatment period, and a follow-up period of up to 12 (± 1) weeks after a single fSCIG 20% infusion (Fig. 1a). The start of the screening period was defined as the date of informed consent to participate in the study.

#### Treatment and Dosing

After admission to the clinic on day − 1 of the study, participants received a single dose of fSCIG 20% on day 1 of the study treatment period, comprising 80 U/g of rHuPH20 and IgG 20%. Different doses of fSCIG 20% were administered in the different treatment arms: in treatment arm 1, IgG 20% was administered at a dose of 0.4 g/kg with in-line warming; in treatment arm 2, IgG 20% was administered at a dose of 1.0 g/kg with in-line warming; and in treatment arm 3, IgG 20% was administered at a dose of 1.0 g/kg with no warming. In all participants, fSCIG 20% was administered using a gradual ramp-up of infusion rates at 10-min intervals. The doses selected aimed to cover the potential dose range for multiple indications for fSCIG 20%. The lower dose of 0.4 g/kg was a representative dose for PIDs, and the higher dose of 1.0 g/kg was representative of dosing levels required for autoimmune neuromuscular diseases (such as chronic inflammatory demyelinating polyradiculoneuropathy or multifocal motor neuropathy, for the treatment of which a fSCIG 10% therapy is under investigation) [21, 22].

A sequential and sentinel dosing design was used as a precaution to ensure optimal tolerability and safety (Fig. 1b). Treatment arms 1, 2, and 3 were dosed consecutively, so that patients receiving in-line warmed IgG 20% at 0.4 g/kg were dosed first, followed by patients receiving in-line warmed IgG 20% at 1.0 g/kg doses. Patients receiving unwarmed IgG 20% at 1.0 g/kg doses were dosed last. In each treatment arm, participants were divided into four subgroups of one, one, two, and four individuals, respectively; the subgroups were dosed sequentially at least 3 days apart to allow for tolerability and safety evaluations prior to initiating dosing of the next subgroup.

### Participant Population

Eligible participants were healthy individuals who fulfilled the following key inclusion criteria: (1) willing to fully comply with the study and able to provide voluntary, informed, written consent to participate; (2) adults aged 19–50 years (inclusive) at the time of informed consent; (3) male, or non-pregnant, non-breastfeeding female of child-bearing potential who agreed to comply with the contraceptive requirements of the protocol or female of non-child-bearing potential; (4) considered healthy by the investigator after finding no evidence of active or chronic disease following a detailed medical and surgical history and a complete physical examination including vital signs, 12-lead electrocardiogram (ECG), blood hematology, blood chemistry, and urinalysis; and (5) had a body mass index (BMI) of 18.0–30.0 kg/m^2^. Exclusion criteria included known or suspected intolerance or hypersensitivity to fSCIG 20%, closely related compounds or any of the stated ingredients, known history of hypersensitivity or severe allergic reaction to blood or blood products, and participation in another clinical study involving immunoglobulin products within 12 months of screening. During the study, the only concomitant medications permitted were hormonal contraceptives for female participants of child-bearing potential, hormone replacement therapy, and over-the-counter medications (if needed and at the discretion of the investigator).

### Administration of Study Infusions

fSCIG 20% was administered at up to two infusion sites sequentially either in the abdomen (middle to upper) or the thigh (left or right), using the same 24-gauge subcutaneous needle set (BD Saf-T-Intima™ Safety System [Becton, Dickinson and Company, Franklin Lakes, NJ, USA]) for both rHuPH20 and IgG 20% using a peristaltic pump (CURLIN 6000 Ambulatory Infusion Pump (Moog Medical, Salt Lake City, UT, USA)). rHuPH20 was administered first via a peristaltic pump at a rate of 120 mL/h/site and at infusion volumes of up to 30 mL/site. Starting within 10 min of completion of rHuPH20 infusion, IgG 20% was administered at infusion volumes of up to 300 mL/site. A maximum infusion rate of 300 mL/h was selected to allow comparison with fSCIG 10%, as well as comparison of this infusion rate between warmed and unwarmed fSCIG 20%. IgG 20% infusion required one or two infusion sites; if two infusion sites were needed, the maximum possible volume was administered at the first infusion site, with the remainder of the total dose subsequently administered into a second site. A flushing step of normal saline was used at the end of IgG 20% infusion to ensure the total dose was administered.

The infusion rate schedule is shown in Table 1. If an infusion rate was reduced or interrupted owing to intolerability, it was reduced to the maximum tolerable rate. In treatment arms 1 and 2, a commercially available in-line warming device was used to warm the infusion tubing used to administer IgG 20%.

**Table 1:** Infusion rate schedule per protocol for all treatment arms

Administration		Rate per infusion site, mL/h	Volume delivered, mL	Accumulative volume for site 1,^a^ mL	Accumulative volume for site 2, mL
rHuPH20	To be infused first	120	N/A^b^	N/A^b^	N/A^b^
IgG 20%	First 10 min	30	5	5	5
	Next 10 min	60	10	15	15
	Next 10 min	120	20	35	35
	Next 10 min	180	30	65	65
	Remainder of infusion	300	N/A^b^	N/A^b^ (up to 300^c^)	N/A^b^ (remainder)

One pump was used sequentially for infusion sites 1 and 2

^a^Participants in treatment arm 1 used only one infusion site. ^b^The volume infused depended on the dose prescribed, as determined by the body weight of the participant. ^c^Excluding the volume of rHuPH20 delivered first

Abbreviations: *IgG*, immunoglobulin G; *N/A,* not applicable; *rHuPH20*, recombinant human hyaluronidase

### Assessments and Study Outcome Measures

Baseline for each assessment was defined as the last available value from screening to day − 1 (fSCIG 20% administration was on day 1).

#### Participant Demographics and Clinical Characteristics

During screening, participant demographics, medical history, and height and weight (for BMI calculation) were collected. Participant medical histories were also collected at day −1 and day 1, and weight was also collected at day −1 (for dose calculation). Details of prior and concomitant medications were collected before and throughout the study, respectively.

#### Tolerability Outcomes

The primary endpoint was the occurrence of tolerability events related to fSCIG 20% per infusion site. A tolerability event was considered to have occurred if the infusion was not interrupted or stopped, and the rate was not reduced owing to any fSCIG 20%-related treatment-emergent adverse event (TEAE).

#### Safety and Immunogenicity Outcomes

Secondary endpoints were the occurrence of TEAEs, clinical laboratory parameters, vital signs, and immunogenicity. TEAEs were defined as any event emerging at or after initiation of fSCIG 20% treatment or any existing event that worsened in intensity or frequency following fSCIG 20% exposure. TEAEs were defined as temporally associated with fSCIG 20% if they occurred within 72 h of completing fSCIG 20% infusion; the investigator determined whether TEAEs were local, systemic, or related to fSCIG 20%. The Common Terminology Criteria for Adverse Events (CTCAE; US Department of Health and Human Services, Washington, DC, USA) [23] was used to assess the severity of adverse events, with grade 1 representing a mild TEAE and grade 5 signifying death related to a TEAE. The Medical Dictionary for Regulatory Activities version 24.1 (MedDRA; Herndon, VA, USA) [24] was used to organize TEAEs into system organ class and preferred term categories. Allergy, catheter leakage, and thromboembolic events were considered as adverse events of special interest.

Clinically significant treatment-emergent changes in clinical laboratory measures and vital signs were also recorded as TEAEs. Blood samples for hemolytic panel were collected at day −1 and on day 4; blood samples for hemoglobin were collected during screening and on day −1 and day 3. Blood samples for serum chemistry, hematology, and urine samples for urinalysis were collected during screening and on day −1 and day 30 (± 3 days). Vital signs were assessed during screening and on day −1, day 1, day 4, and day 30 (± 3 days), and at the end of the study; 12-lead ECG was also performed during screening and on day 1 within 1 h of fSCIG 20% dosing.

Immunogenicity was determined by the occurrence of binding and neutralizing antibodies to rHuPH20. Blood samples for binding anti-rHuPH20 antibodies were collected on day −1 and day 30 (± 3 days) post-dose, and at the end of the study; post-dose samples with anti-rHuPH20 antibody titers ≥ 1:160 were analyzed for the presence of neutralizing antibodies.

There were also subcutaneous administration endpoints that represented supportive tolerability and safety measures and consisted of the maximum tolerable infusion rate achieved, total volume infused, and infusion duration (all per infusion site).

### Statistical Analysis

All statistical analyses were descriptive; no statistical hypothesis was tested. The planned total sample size for this study was 24 participants (8 per treatment arm), with a minimum of 18 individuals expected to complete the study. This sample size was considered adequate for assessing the tolerability and safety of fSCIG 20% based on clinical judgment. Statistical Analysis Software (SAS; SAS Institute, Cary, NC, USA) version 9.4 or higher was used to conduct all statistical analyses.

## Results

### Participant Disposition and Baseline Demographics

In total, 24 participants were enrolled in the study, eight in each treatment arm. Twenty-three participants (95.8%) completed the study; one participant received the planned fSCIG 20% dose but discontinued during the follow-up period owing to a family emergency. Overall, the mean (standard deviation (SD)) age of participants was 39.0 (7.3) years (Table 2). Most participants were White (23 participants (95.8%)), and 13 individuals (54.2%) were male. Mean (SD) BMI was 24.9 (3.3) kg/m^2^. Baseline demographic characteristics were broadly similar between treatment arms, except for the proportions of male and female participants. No participants received prior medications; four participants (16.7%) received concomitant medications of contraceptives, iron, or acetaminophen/paracetamol, which were not expected to affect endpoint assessments.

**Table 2:** Baseline participant characteristics

Parameter	Treatment arm 1 fSCIG 20% with warmed 0.4 g/kg IgG 20% (*n* = 8)	Treatment arm 2 fSCIG 20% with warmed 1.0 g/kg IgG 20% (*n* = 8)	Treatment arm 3 fSCIG 20% with unwarmed 1.0 g/kg IgG 20% (*n* = 8)	All participants (*N* = 24)
Age,^a^ years				
Mean (SD)	42.6 (4.6)	39.4 (4.1)	34.9 (10.1)	39.0 (7.3)
Median (range)	43.0 (36.0–49.0)	39.5 (33.0–46.0)	33.5 (23.0–49.0)	40.5 (23.0–49.0)
Sex, *n* (%)				
Male	3 (37.5)	6 (75.0)	4 (50.0)	13 (54.2)
Female	5 (62.5)	2 (25.0)	4 (50.0)	11 (45.8)
Ethnicity, *n* (%)				
Hispanic or Latino	8 (100.0)	7 (87.5)	8 (100.0)	23 (95.8)
Not Hispanic or Latino	0 (0.0)	1 (12.5)	0 (0.0)	1 (4.2)
Race, *n* (%)				
White	8 (100.0)	7 (87.5)	8 (100.0)	23 (95.8)
Black or African American	0 (0.0)	1 (12.5)	0 (0.0)	1 (4.2)
Weight, kg				
Mean (SD)	70.9 (6.8)	68.1 (14.2)	70.9 (14.3)	70.0 (11.8)
Median (range)	70.2 (61.7–81.0)	64.1 (52.4–92.0)	66.9 (49.6–91.3)	69.1 (49.6–92.0)
BMI^b^, kg/m^2^				
Mean (SD)	25.9 (2.4)	23.3 (4.0)	25.6 (3.3)	24.9 (3.3)
Median (range)	25.8 (21.9–30.00)	22.4 (19.0–30.0)	25.3 (21.7–29.2)	25.0 (19.0–30.0)
BMI 18– < 25 kg/m^2^, *n*	3	5	4	12
BMI 25–30 kg/m^2^, *n*	5	3	4	12

^a^Age at the date of informed consent signature. ^b^BMI was calculated as weight (kg) ÷ height^2^ (m^2^) using height and weight values from the screening visit

Abbreviations: *BMI*, body mass index; *fSCIG 20%, *hyaluronidase-facilitated subcutaneous immunoglobulin 20%; *IgG*, immunoglobulin G; *SD*, standard deviation

### Tolerability

Infusions into the first infusion site (mean total IgG 20% volume, 141.3, 291.3, and 293.8 mL in treatment arms 1, 2, and 3, respectively) were tolerable in all participants (*n* = 24). For participants requiring a second infusion site (*n* = 0, 4, and 7 participants in treatment arms 1, 2, and 3, respectively), all infusions into the second infusion site were also tolerated by all participants (*n* = 11; mean total IgG 20% volume, 97.5 and 70.0 mL in treatment arms 2 and 3, respectively). An infusion was interrupted in one participant in treatment arm 3 owing to a mechanical issue (kinking of catheter tubing), which led to a high-pressure in-line catheter that triggered a pump alarm. The catheter was repositioned, the pressure returned to normal, and the infusion was restarted at 30 mL/h. The interruption was neither TEAE- nor tolerability-related.

### Safety

#### Adverse Events

In total, 107 TEAEs were reported in 24 participants (100.0%), all of which were temporally associated with fSCIG 20% infusion (Table 3). Of these, 105 (occurring in 24 participants (100.0%)) were considered related to fSCIG 20%. There were 102 local TEAEs reported in 24 participants (100.0%) and 5 systemic TEAEs occurring in 4 participants (16.7%). All TEAEs were deemed to be mild in severity and were recovered/resolved by the end of the study. The duration of TEAEs ranged from 0.17 h to 8 days. One participant in treatment arm 1 had a TEAE of special interest (catheter leakage post-infusion). There were no deaths, severe, or serious adverse events reported, and no participant discontinued the study owing to TEAEs. The occurrence of TEAEs was broadly similar between BMI 18–< 25 and 25–30 kg/m^2^ groups (Supplemental Table 1)

**Table 3:** Overview of TEAEs

Parameter	Treatment arm 1 fSCIG 20% with warmed 0.4 g/kg IgG 20% (*n* = 8)		Treatment arm 2 fSCIG 20% with warmed 1.0 g/kg IgG 20% (*n* = 8)		Treatment arm 3 fSCIG 20% with unwarmed 1.0 g/kg IgG 20% (*n* = 8)		All participants (*N* = 24)	
	Number of participants (%)	Number of events	Number of participants (%)	Number of events	Number of participants (%)	Number of events	Number of participants (%)	Number of events
Any TEAE	8 (100.0)	27	8 (100.0)	34	8 (100.0)	46	24 (100.0)	107
TEAEs related to fSCIG 20%	8 (100.0)	25	8 (100.0)	34	8 (100.0)	46	24 (100.0)	105
Temporally associated TEAEs (within 72 h of the infusion)	8 (100.0)	27	8 (100.0)	34	8 (100.0)	46	24 (100.0)	107
Serious TEAEs	0 (0.0)	0	0 (0.0)	0	0 (0.0)	0	0 (0.0)	0
TEAEs leading to study discontinuation	0 (0.0)	0	0 (0.0)	0	0 (0.0)	0	0 (0.0)	0
Local TEAEs	8 (100.0)	24	8 (100.0)	34	8 (100.0)	44	24 (100.0)	102
Systemic TEAEs	2 (25.0)	3	0 (0.0)	0	2 (25.0)	2	4 (16.7)	5
CTCAE grade 1 TEAEs	8 (100.0)	27	8 (100.0)	34	8 (100.0)	46	24 (100.0)	107
TEAEs of special interest	1 (12.5)	1^a^	0 (0.0)	0	0 (0.0)	0	1 (4.2)	1

A TEAE was defined as any adverse event that started at or after initiation of fSCIG 20% treatment

^a^The TEAE of special interest was catheter leakage

Abbreviations: *CTCAE*, Common Terminology Criteria for Adverse Events; *fSCIG 20%*, hyaluronidase-facilitated subcutaneous immunoglobulin 20%; *IgG*, immunoglobulin G; *TEAE*, treatment-emergent adverse event

**Fig. 2:**
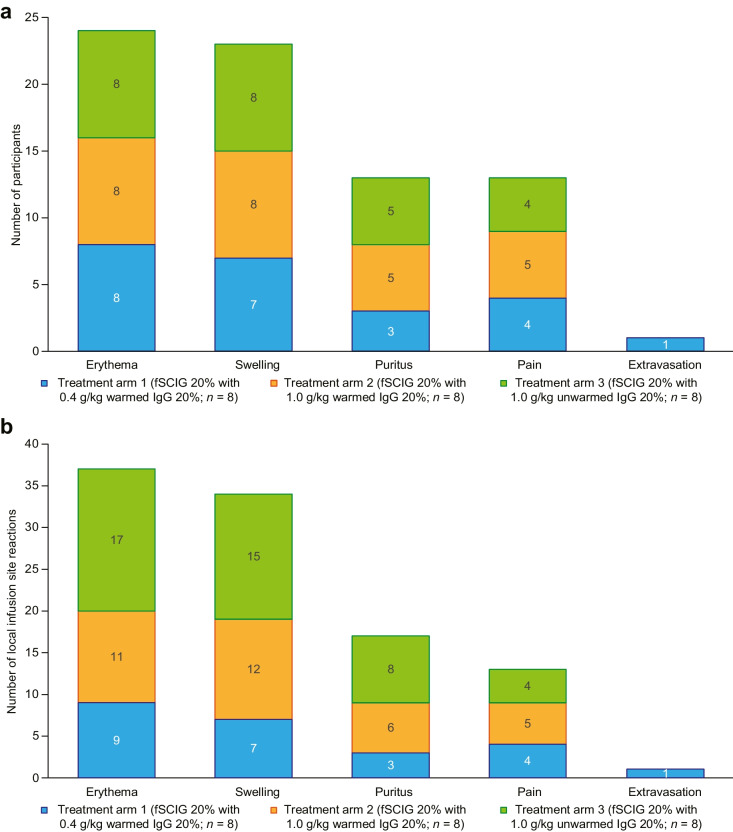
Number of participants who experienced local infusion site reactions **a** and number of local infusion site reactions reported **b**. Events were assessed per infusion site, and more than one of the same type of event may have occurred per infusion site on different days. Abbreviations: *fSCIG 20%*, hyaluronidase-facilitated subcutaneous immunoglobulin 20%; *IgG*, immunoglobulin G

**Fig. 3:**
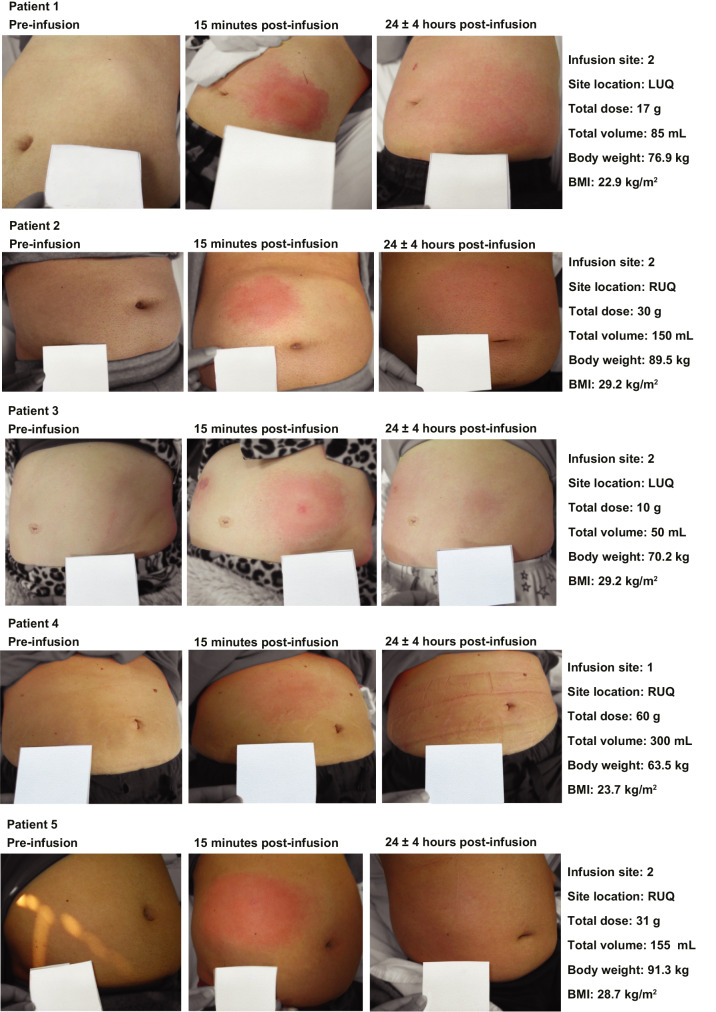
Representative images of local infusion site reactions. Images are shown for participants in treatment arm 3. Details of dose and volume are per site. Abbreviations: *BMI*, body mass index; *LUQ*, left upper quadrant; *RUQ*, right upper quadrant

In treatment arm 1, there were 27 TEAEs in 8 participants (100.0%), of which 25 TEAEs were considered related to fSCIG 20%. In treatment arms 2 and 3, there were 34 TEAEs in 8 participants (100.0%), and 46 TEAEs in 8 participants (100.0%), respectively, all of which were considered related to fSCIG 20%. Most TEAEs were infusion site-related (102 TEAEs reported in 24 participants (100.0%); Table 4). The most frequent TEAEs reported in more than 50% of participants were infusion site erythema (37 TEAEs in 24 participants (100.0%)), infusion site swelling (34 TEAEs in 23 participants (95.8%)), infusion site pruritis (17 TEAEs in 13 participants (54.2%)), and infusion site pain (13 TEAEs in 13 participants (54.2%)). TEAEs by treatment arm are shown in Fig. 2 and infusion site reactions in Fig. 3.

**Table 4:** Summary of TEAEs by system organ class and preferred term

System organ class Preferred term	Treatment arm 1 fSCIG 20% with warmed 0.4 g/kg IgG 20% (*n* = 8)		Treatment arm 2 fSCIG 20% with warmed 1.0 g/kg IgG 20% (*n* = 8)		Treatment arm 3 fSCIG 20% with unwarmed 1.0 g/kg IgG 20% (*n* = 8)		All participants (*n* = 24)	
	Number of participants (%)	Number of events	Number of participants (%)	Number of events	Number of participants (%)	Number of events	Number of participants (%)	Number of events
Gastrointestinal disorders	1 (12.5)	2	0 (0.0)	0	0 (0.0)	0	1 (4.2)	2
Diarrhea	1 (12.5)	1	0 (0.0)	0	0 (0.0)	0	1 (4.2)	1
Nausea	1 (12.5)	1	0 (0.0)	0	0 (0.0)	0	1 (4.2)	1
General disorders and administration site conditions	8 (100.0)	24	8 (100.0)	34	8 (100.0)	44	24 (100.0)	102
Infusion site erythema	8 (100.0)	9	8 (100.0)	11	8 (100.0)	17	24 (100.0)	37
Infusion site swelling	7 (87.5)	7	8 (100.0)	12	8 (100.0)	15	23 (95.8)	34
Infusion site pruritus	3 (37.5)	3	5 (62.5)	6	5 (62.5)	8	13 (54.2)	17
Infusion site pain	4 (50.0)	4	5 (62.5)	5	4 (50.0)	4	13 (54.2)	13
Infusion site extravasation	1 (12.5)	1	0 (0.0)	0	0 (0.0)	0	1 (4.2)	1
Nervous system disorders	1 (12.5)	1	0 (0.0)	0	2 (25.0)	2	3 (12.5)	3
Headache	1 (12.5)	1	0 (0.0)	0	1 (12.5)	1	2 (8.3)	2
Dizziness	0 (0.0)	0	0 (0.0)	0	1 (12.5)	1	1 (4.2)	1

A TEAE was defined as any adverse event that started at or after initiation of fSCIG 20% treatment. MedDRA version 24.1 was used to organize adverse events into system organ class and preferred term

Abbreviations: *fSCIG 20%*, hyaluronidase-facilitated subcutaneous immunoglobulin 20%; *IgG*, immunoglobulin G; *MedDRA*, Medical Dictionary for Regulatory Activities; *TEAE*, treatment-emergent adverse event

#### Clinical Laboratory Parameters and Vital Signs

In general, there were no clinically meaningful changes from baseline or apparent differences between treatment arms in clinical laboratory parameters or vital signs. No participants had a clinically significant abnormal ECG result. No changes in clinical laboratory parameters, vital signs, or ECG results were reported as TEAEs.

#### Immunogenicity

All participants tested negative for binding anti-rHuPH20 antibodies (titer < 1:160). Testing for neutralizing anti-rHuPH20 antibodies was therefore not performed.

#### Supportive Tolerability and Safety Measures

In all participants, the maximum tolerable infusion rate for infusion site 1 was 300 mL/h/site (Table 5). The mean total IgG 20% volume infused into infusion site 1 was 141.3, 291.3, and 293.8 mL in treatment arms 1, 2, and 3, respectively. For infusion sites 1 and 2 combined, total volume infused and infusion duration varied between treatment arms, in line with the associated difference in fSCIG 20% dose. The mean total IgG 20% volume infused was 141.3, 340.0, and 355.0 mL in treatment arms 1, 2, and 3, respectively. The mean infusion duration was 65.0, 124.9, and 139.3 min, for treatment arms 1, 2, and 3, respectively. Actual total IgG 20% dose, total IgG 20%, and rHuPH20 volumes and total infusion durations were greater in the 18–24 than the 25–30 kg/m^2^ BMI group (Supplemental Table 2).

**Table 5:** Summary of dosing and infusion parameters

Parameter, mean (SD)	Treatment arm 1 fSCIG 20% with warmed 0.4 g/kg IgG 20%		Treatment arm 2 fSCIG 20% with warmed 1.0 g/kg IgG 20%		Treatment arm 3 fSCIG 20% with unwarmed 1.0 g/kg IgG 20%	
	*n*	Value	*n*	Value	*n*	Value
IgG 20% maximum tolerable infusion rate,^a^ number of participants (%)						
300 mL/h	8	8 (100.0)	8	8 (100.0)	8	8 (100.0)
Actual total IgG 20% dose,^b^ mean (SD), g	8	28.3 (2.7)	8	68.0 (14.2)	8	71.0 (14.2)
Total IgG 20% volume infused,^b^ mean (SD), mL	8	141.3 (13.3)	8	340.0 (71.0)	8	355.0 (71.1)
Infusion site 1	8	141.3 (13.3)	8	291.3 (14.3)	8	293.8 (17.7)
Infusion site 2^c^	0	N/A	4	97.5 (51.7)	7	70.0 (61.6)
Total rHuPH20 volume infused,^b^ mean (SD), mL	8	14.3 (1.3)	8	34.3 (7.1)	8	35.8 (7.2)
Infusion site 1	8	14.3 (1.3)	8	29.3 (1.4)	8	29.4 (1.8)
Infusion site 2^c^	0	N/A	4	10.0 (5.2)	7	7.3 (6.2)
Total infusion duration,^b^ mean (SD), minutes	8	65.0 (5.2)	8	124.9 (32.0)	8	139.3 (26.5)
Infusion site 1	8	65.0 (5.2)	8	98.8 (3.5)	8	102.3 (5.9)
Infusion site 2^c^	0	N/A	4	52.3 (14.4)	7	42.3 (20.3)

^a^Data shown for infusion site 1. ^b^Combined data for infusion sites 1 and 2. ^c^A total of 0, 4, and 7 patients used a second infusion site in treatment arms 1, 2, and 3, respectively

Abbreviations: *fSCIG 20%*, hyaluronidase-facilitated immunoglobulin 20%; *IgG*, immunoglobulin G; *N/A*, not applicable; *rHuPH20*, recombinant human hyaluronidase; *SD*, standard deviation

## Discussion

This phase 1, open-label study assessed the tolerability and safety profile of fSCIG 20%, which combines the benefits of both IVIG and SCIG therapies while offering smaller infusion volumes for the same dose than less-concentrated treatments, less frequent infusions, and reduced infusion duration and needle sticks than with conventional SCIG. Infusions of warmed and unwarmed fSCIG 20% were well-tolerated in all participants at rates of up to 300 mL/h/site, with favorable safety profiles. These findings are of particular importance when considering the potential benefits to patients offered by fSCIG 20% in terms of increased convenience and reduced treatment burden and the potential opportunities for individualized treatment and improved patient convenience.

Assessing the safety, tolerability, and infusion parameters of immunoglobulin therapies under development in healthy participants provides critical data when determining an optimal administration strategy and in understanding their potential safety and tolerability in future studies in larger patient populations. The tolerability of fSCIG 20% infusion seen in this analysis was favorable, despite higher infusion rates and increased concentration when compared with conventional SCIG 20% [25–27] and fSCIG 10% [28], respectively. The maximum infusion rate of 300 mL/h/site observed is the same as the recommended maximum infusion rate with fSCIG 10% [11, 29] and 5–12 times the maximum recommended infusion rate of conventional SCIG 20% therapies (25–60 mL/h/site) [14, 17, 18, 30]. These results indicate that fSCIG 20% may allow for shortened infusion times (mean total duration 124.9 and 139.3 min in warmed and unwarmed 1.0 g/kg treatment arms, respectively) in patients requiring high-volume infusions.

All participants experienced TEAEs; nearly all TEAEs were related to fSCIG 20% infusions, and all were CTCAE grade 1, indicating that they were mild in severity. In keeping with results from the current analysis, previous studies of fSCIG 10% and conventional SCIG 20% reported that TEAEs were most frequently mild in severity [25–27, 29–33] with low rates of treatment-related serious adverse events (reported for < 1.0% of patients) [22, 25–27, 29–38]. One TEAE, a single event of catheter leakage, was of special interest as defined in the protocol; however, the leakage was small. All TEAEs were recovered or resolved by the end of the study.

Nearly all TEAEs were local infusion site reactions, and all participants experienced local infusion site reactions in this study, most frequently infusion site erythema and swelling, which is consistent with administration of conventional SCIG. Local infusion site reactions most often reported by previous patient studies of fSCIG 10% and conventional SCIG 20% include infusion site erythema, pain, pruritis, swelling, inflammation, and discomfort [25–27, 30, 32, 34, 38–41]. No participants tested positive for binding anti-rHuPH20 antibodies after a single dose of fSCIG 20%.

Warming of IgG 20% may potentially reduce the viscosity of concentrated IgG therapies and improve tolerability. In this study, all infusions were tolerated, and a maximum tolerable infusion rate of 300 mL/h/site was achieved in all treatment arms, with and without inline warming. Although the unwarmed arm at the 1 g/kg dose level appeared to be associated with a slightly greater number of TEAEs, all TEAEs were mild in severity. It should also be noted that seven of the eight patients required two infusion sites owing to dose. Based on the findings of this study, and considering the complexity and reduced patient convenience of using a warming device (important factors in patient preference for SCIG therapies [42]), administration of fSCIG 20% without in-line warming is the preferred option for future clinical development.

The strengths of this study include the application of the sequential and sentinel study design to ensure optimal tolerability and safety assessment and comparison of warmed and unwarmed treatment. This study also included healthy participants, who are often included in studies of monoclonal antibodies [43–46], although their inclusion in studies of IgRT has been uncommon. The normal endogenous IgG levels in healthy participants limit the usefulness of pharmacokinetic evaluations, which are often endpoints in studies of IgRT. However, studies in healthy participants can be useful for the evaluation of tolerability, safety, and infusion parameters with immunoglobulin therapies and allow for fast, convenient, and homogenous evaluation of IgRT.

This study was limited by a relatively small sample size and inclusion of only a small number of participants who received unwarmed fSCIG 20%. The study also included healthy participants who may have not received prior immunoglobulin therapy and received only a single dose of fSCIG 20%; the frequency and characteristics of adverse events in these participants may, therefore, be different in some cases to those of patients who regularly receive IgRT. Nonetheless, results support further evaluation of fSCIG 20% in patients with PIDs. Further clinical development will aim to identify the optimal method of administration for fSCIG 20% that provides the best patient experience, reduces treatment burden, and shortens infusion times compared with existing IgRT, as well as allowing for individualized treatment regimens.

## Conclusions

fSCIG 20%, both warmed and unwarmed, was well-tolerated and had a favorable safety profile at doses of up to 1 g/kg/infusion and infusion rates of up to 300 mL/h/site. There were no substantial differences between treatment arms in tolerability, TEAEs, or immunogenicity, suggesting minimal impact of in-line warming of IgG 20% on the tolerability and safety profile of fSCIG 20%. The pharmacokinetics, safety, tolerability, and immunogenicity of unwarmed fSCIG 20% will be further investigated in patients with PIDs.

### Supplementary Information

Below is the link to the electronic supplementary material.

Supplementary file1 (DOCX 30 KB)

## Data Availability

Data pertaining to this study and the associated study protocol may be found at ClinicalTrials.gov (identifier: NCT05059977). The data sets, including the redacted study protocol, redacted statistical analysis plan, and individual participant data supporting the results reported in this article, will be made available within 3 months from initial request to researchers who provide a methodologically sound proposal. The data will be provided after its de-identification, in compliance with applicable privacy laws, data protection, and requirements for consent and anonymization.

## References

[1] Misbah S, Sturzenegger M, Borte M, Shapiro R, Wasserman R, Berger M (2009). Subcutaneous immunoglobulin: opportunities and outlook. Clin Exp Immunol.

[2] Perez E, Orange J, Bonilla F, Chinen J, Chinn I, Dorsey M (2017). Update on the use of immunoglobulin in human disease: a review of evidence. J Allergy Clin Immunol.

[3] Bonilla F, Khan D, Ballas Z, Chinen J, Frank M, Hsu J (2015). Practice parameter for the diagnosis and management of primary immunodeficiency. J Allergy Clin Immunol.

[4] Tangye S, Al-Herz W, Bousfiha A, Cunningham-Rundles C, Franco J, Holland S (2022). Human inborn errors of immunity: 2022 update on the classification from the International Union of Immunological Societies expert committee. J Clin Immunol.

[5] Abolhassani H, Azizi G, Sharifi L, Yazdani R, Mohsenzadegan M, Delavari S (2020). Global systematic review of primary immunodeficiency registries. Expert Rev Clin Immunol.

[6] Demirdag Y, Gupta S (2021). Update on infections in primary antibody deficiencies. Front Immunol.

[7] Epland K, Suez D, Paris K (2022). A clinician's guide for administration of high-concentration and facilitated subcutaneous immunoglobulin replacement therapy in patients with primary immunodeficiency diseases. Allergy Asthma Clin Immunol.

[8] Krivan G, Jolles S, Granados E, Paolantonacci P, Ouaja R, Cisse O (2017). New insights in the use of immunoglobulins for the management of immune deficiency (PID) patients. Am J Clin Exp Immunol.

[9] Espanol T, Prevot J, Drabwell J, Sondhi S, Olding L (2014). Improving current immunoglobulin therapy for patients with primary immunodeficiency: quality of life and views on treatment. Patient Prefer Adherence.

[10] Mohamed A, Kilambi V, Luo M, Iyer R, Li-McLeod J (2012). Patient and parent preferences for immunoglobulin treatments: a conjoint analysis. J Med Econ.

[11] Baxalta US Inc. HYQVIA (immune globulin infusion 10% [human] with recombinant human hualuronidase) solution for subcutaneous administration. Prescribing information 2021. Available from: https://www.shirecontent.com/PI/PDFs/HYQVIA_USA_ENG.pdf. Accessed 28 Apr 2023

[12] European MA. HyQvia 100 mg/ml solution for infusion for subcutaneous use. Summary of product characteristics 2022. Available from: https://www.medicines.org.uk/emc/product/9197/smpc#gref. Accessed 28 Apr 2023

[13] Wasserman R (2017). Recombinant human hyaluronidase-facilitated subcutaneous immunoglobulin infusion in primary immunodeficiency diseases. Immunotherapy.

[14] US Food and Drug Administration. CUVITRU, immune globulin subcutaneous (human), 20% solution. Prescribing information 2021. Available from: https://www.fda.gov/media/100531/download. Accessed 28 Apr 2023

[15] European MA. Cuvitru 200 mg/ml solution for subcutaneous injection 2022. Available from: https://www.medicines.org.uk/emc/product/9191/smpc#gref. Accessed 28 Apr 2023

[16] European Medicines Agency. Hizentra 200 mg/ml solution for subcutaneous injection. Summary of product characteristics 2022. Available from: https://www.medicines.org.uk/emc/product/4643/smpc#gref. Accessed 23 Jan 2023

[17] CSL Behring LLC. HIZENTRA® immune globulin subcutaneous (human) 20% liquid. Prescribing information 2022. Available from: https://labeling.cslbehring.com/PI/US/Hizentra/EN/Hizentra-Prescribing-Information.pdf. Accessed 21 Apr 2023

[18] Grifols Therapeutics LLC. XEMBIFY (immune globulin subcutaneous, human – klhw) 20% solution. Prescribing information 2020. Available from: https://www.xembify.com/documents/2600280/0/Xembify+Prescribing+Information.pdf/c768aaca-68b0-4212-9625-59d2a92549f9?t=1641996720258. Accessed 22 Mar 2023

[19] Jolles S, Orange J, Gardulf A, Stein M, Shapiro R, Borte M (2015). Current treatment options with immunoglobulin G for the individualization of care in patients with primary immunodeficiency disease. Clin Exp Immunol.

[20] Leidenmuhler P, Hofinghoff J, Haider N, Brachtl G, Weiller M, Bilic I (2023). In-line warming reduces in-line pressure of subcutaneous infusion of concentrated immunoglobulins. Drug Deliv Transl Res.

[21] Bril V, Hadden RD, Brannagan III TH, Bar M, Chroni E, Rejdak K, et al. Hyaluronidase‐facilitated subcutaneous immunoglobulin 10% as maintenance therapy for chronic inflammatory demyelinating polyradiculoneuropathy: the ADVANCE‐CIDP 1 randomized controlled trial. J Peripher Nerv Syst. 2023;28(3):436–49. 10.1111/jns.12573 37314318

[22] Al-Zuhairy A, Jakobsen J, Andersen H, Sindrup S, Markvardsen L. Randomized trial of facilitated subcutaneous immunoglobulin in multifocal motor neuropathy. Eur J Neurol. 2019;26(10):1289–e82. 10.1111/ene.13978 31021036

[23] U.S. DoHaHS. Common terminology criteria for adverse events (CTCAE), version 5.0 2017. Available from: https://ctep.cancer.gov/protocoldevelopment/electronic_applications/docs/ctcae_v5_quick_reference_5x7.pdf. Accessed 30 Nov 2022

[24] Medical DfRA. Introductory guide MedDRA version 24.1 2021. Available from: https://admin.meddra.org/sites/default/files/guidance/file/000594_intguide_%2024_1.pdf. Accessed 1 Dec 2022

[25] Suez D, Stein M, Gupta S, Hussain I, Melamed I, Paris K (2016). Efficacy, safety, and pharmacokinetics of a novel human immune globulin subcutaneous, 20 % in patients with primary immunodeficiency diseases in North America. J Clin Immunol.

[26] Borte M, Krivan G, Derfalvi B, Marodi L, Harrer T, Jolles S (2017). Efficacy, safety, tolerability and pharmacokinetics of a novel human immune globulin subcutaneous, 20%: a Phase 2/3 study in Europe in patients with primary immunodeficiencies. Clin Exp Immunol.

[27] Suez D, Krivan G, Jolles S, Stein M, Gupta S, Paris K (2019). Safety and tolerability of subcutaneous immunoglobulin 20% in primary immunodeficiency diseases from two continents. Immunotherapy.

[28] Ellerbroek T, Witte T, Lougaris V, Chen J, Nagy A, McCoy B, et al. (90) Final analysis of long-term safety of facilitated subcutaneous immunoglobulin across the age range: results from a postauthorization study. J Clin Immunol. 2022;42(Suppl 1):S49–50.

[29] Wasserman R, Melamed I, Stein M, Gupta S, Puck J, Engl W (2012). Recombinant human hyaluronidase-facilitated subcutaneous infusion of human immunoglobulins for primary immunodeficiency. J Allergy Clin Immunol.

[30] Jolles S, Borte M, Nelson R, Rojavin M, Bexon M, Lawo J (2014). Long-term efficacy, safety, and tolerability of Hizentra® for treatment of primary immunodeficiency disease. Clin Immunol.

[31] Keith P, Cowan J, Kanani A, Kim H, Lacuesta G, Lee J (2022). Transitioning subcutaneous immunoglobulin 20% therapies in patients with primary and secondary immunodeficiencies: Canadian real-world study. Allergy Asthma Clin Immunol.

[32] Viallard J, Agape P, Barlogis V, Cozon G, Faure C, Fouyssac F (2016). Treatment with Hizentra in patients with primary and secondary immunodeficiencies: a real-life, non-interventional trial. BMC Immunol.

[33] Sleasman J, Lumry W, Hussain I, Wedner H, Harris J, Courtney K (2019). Immune globulin subcutaneous, human - klhw 20% for primary humoral immunodeficiency: an open-label, phase III study. Immunotherapy.

[34] Wasserman R, Melamed I, Stein M, Engl W, Sharkhawy M, Leibl H (2016). Long-term tolerability, safety, and efficacy of recombinant human hyaluronidase-facilitated subcutaneous infusion of human immunoglobulin for primary immunodeficiency. J Clin Immunol.

[35] Fasshauer M, Borte M, Bitzenhofer M, Pausch C, Pittrow D, Park M, et al. Real-world utilization, safety and patient experience of 20% subcutaneous immunoglobulin in patients with primary immunodeficiencies: final data from the CORE study. Poster presented at: 20th Biennial Meeting of The European Society for Immunodeficiencies (ESID), October 12–15, 2022, Gothenburg, Sweden.

[36] Jolles S, Bernatowska E, de Gracia J, Borte M, Cristea V, Peter H (2011). Efficacy and safety of Hizentra® in patients with primary immunodeficiency after a dose-equivalent switch from intravenous or subcutaneous replacement therapy. Clin Immunol.

[37] Hagan J, Fasano M, Spector S, Wasserman R, Melamed I, Rojavin M (2010). Efficacy and safety of a new 20% immunoglobulin preparation for subcutaneous administration, IgPro20, in patients with primary immunodeficiency. J Clin Immunol.

[38] van Schaik I, Mielke O, Bril V, van Geloven N, Hartung H, Lewis R (2019). Long-term safety and efficacy of subcutaneous immunoglobulin IgPro20 in CIDP: PATH extension study. Neurol Neuroimmunol Neuroinflamm.

[39] Borte M, Hanitsch LG, Mahlaoui N, Fasshauer M, Huscher D, Speletas M, et al. Facilitated subcutaneous immunoglobulin treatment in patients with immunodeficiencies: the FIGARO study. J Clin Immunol 2023;1–13. 10.1007/s10875-023-01470-2 PMC10088636 37036560

[40] Vultaggio A, Azzari C, Ricci S, Martire B, Palladino V, Gallo V (2018). Biweekly Hizentra® in primary immunodeficiency: a multicenter, observational cohort study (IBIS). J Clin Immunol.

[41] Santamaria M, Neth O, Douglass J, Krivan G, Kobbe R, Bernatowska E (2022). A multi-center, open-label, single-arm trial to evaluate the efficacy, pharmacokinetics, and safety and tolerability of IGSC 20% in subjects with primary immunodeficiency. J Clin Immunol.

[42] Overton P, Shalet N, Somers F, Allen J (2021). Patient preferences for subcutaneous versus intravenous administration of treatment for chronic immune system disorders: a systematic review. Patient Prefer Adherence.

[43] Blank A, Hohmann N, Dettmer M, Manka-Stuhlik A, Mikus G, Stoll F (2022). First-in-human, randomized, double-blind, placebo-controlled, dose escalation trial of the anti-herpes simplex virus monoclonal antibody HDIT101 in healthy volunteers. Clin Transl Sci.

[44] Joseph D, Thoma C, Haeufel T, Li X (2022). Assessment of the pharmacokinetics and safety of spesolimab, a humanised anti-interleukin-36 receptor monoclonal antibody, in healthy non-Japanese and Japanese Subjects: results from phase I clinical studies. Clin Pharmacokinet.

[45] Nowotny B, Thomas D, Schwers S, Wiegmann S, Prange W, Yassen A (2022). First randomized evaluation of safety, pharmacodynamics, and pharmacokinetics of BAY 1831865, an antibody targeting coagulation factor XI and factor XIa, in healthy men. J Thromb Haemost.

[46] Raja S, Guptill J, Juel V, Walter E, Cohen-Wolkowiez M, Hill H (2022). First-in-human clinical trial to assess the safety, tolerability and pharmacokinetics of single doses of NTM-1633, a novel mixture of monoclonal antibodies against botulinum toxin E. Antimicrob Agents Chemother.

