# Supplementary material for: A Phase 1 Open-Label Study to Assess the Tolerability, Safety, and Immunogenicity of Hyaluronidase-Facilitated Subcutaneous Immunoglobulin 20% in Healthy Adults
Source: J Clin Immunol. 2023 Dec 22;44(1):28. doi: 10.1007/s10875-023-01632-2 (PMC10739571; doi:10.1007/s10875-023-01632-2)
Supplement: Supplementary file 1 — Supplementary file1 (DOCX 30 KB) [file 10875_2023_1632_MOESM1_ESM.docx]

# Supplementary material

## Supplemental Table 1 Overview of TEAEs by BMI group

| **Parameter** | **Treatment arm 1**  **fSCIG 20% with warmed 0.4 g/kg IgG 20%** | | | | **Treatment arm 2**  **fSCIG 20% with warmed 1.0 g/kg IgG 20%** | | | | **Treatment arm 3**  **fSCIG 20% with unwarmed 1.0 g/kg IgG 20%** | | | |
| --- | --- | --- | --- | --- | --- | --- | --- | --- | --- | --- | --- | --- |
|  | **BMI 18–<25 kg/m^2^  (*n* = 3)** | | **BMI 25–30 kg/m^2^  (*n* = 5)** | | **BMI 18–<25 kg/m^2^  (*n* = 5)** | | **BMI 25–30 kg/m^2^  (*n* = 3)** | | **BMI 18-<25 kg/m^2^  (*n* = 4)** | | **BMI 25–30 kg/m^2^  (*n* = 4)** | |
|  | **Number of participants (%)** | **Number of events** | **Number of participants (%)** | **Number of events** | **Number of participants (%)** | **Number of events** | **Number of participants (%)** | **Number of events** | **Number of participants (%)** | **Number of events** | **Number of participants (%)** | **Number of events** |
| **Any TEAE** | 3 (100.0) | 8 | 5 (100.0) | 19 | 5 (100.0) | 18 | 3 (100.0) | 16 | 4 (100.0) | 24 | 4 (100.0) | 22 |
| **TEAEs related to fSCIG 20%** | 3 (100.0) | 8 | 5 (100.0) | 17 | 5 (100.0) | 18 | 3 (100.0) | 16 | 4 (100.0) | 24 | 4 (100.0) | 22 |
| **Temporally associated TEAEs (within 72 hours of the infusion)** | 3 (100.0) | 8 | 5 (100.0) | 19 | 5 (100.0) | 18 | 3 (100.0) | 16 | 4 (100.0) | 24 | 4 (100.0) | 22 |
| **Serious TEAEs** | 0 (0.0) | 0 | 0 (0.0) | 0 | 0 (0.0) | 0 | 0 (0.0) | 0 | 0 (0.0) | 0 | 0 (0.0) | 0 |
| **TEAEs leading to study discontinuation** | 0 (0.0) | 0 | 0 (0.0) | 0 | 0 (0.0) | 0 | 0 (0.0) | 0 | 0 (0.0) | 0 | 0 (0.0) | 0 |
| **Local TEAEs** | 3 (100.0) | 8 | 5 (100.0) | 16 | 5 (100.0) | 18 | 3 (100.0) | 16 | 4 (100.0) | 22 | 4 (100.0) | 22 |
| **Systemic TEAEs** | 0 (0.0) | 0 | 2 (40.0) | 3 | 0 (0.0) | 0 | 0 (0.0) | 0 | 2 (50.0) | 2 | 0 (0.0) | 0 |
| **CTCAE grade 1 TEAEs** | 3 (100.0) | 8 | 5 (100.0) | 19 | 5 (100.0) | 18 | 3 (100.0) | 16 | 4 (100.0) | 24 | 4 (100.0) | 22 |
| **TEAEs of special interest** | 0 (0.0) | 0 | 1 (20.0) | 1^a^ | 0 (0.0) | 0 | 0 (0) | 0 | 0 (0.0) | 0 | 0 (0.0) | 0 |

A TEAE was defined as any adverse event that started at or after initiation of fSCIG 20% treatment.

^a^The TEAE of special interest was catheter leakage.

Abbreviations: *BMI*, body mass index; *CTCAE*, Common Terminology Criteria for Adverse Events; *fSCIG 20%*, facilitated subcutaneous immunoglobulin 20%; *IgG*, immunoglobulin G; *TEAE*, treatment-emergent adverse event

## Supplemental Table 2 Dosing and infusion parameters by BMI group

| **Parameter, mean (SD)** | **Treatment arm 1**  **fSCIG 20% with warmed 0.4 g/kg IgG 20%** | | | | **Treatment arm 2**  **fSCIG 20% with warmed 1.0 g/kg IgG 20%** | | | | **Treatment arm 3**  **fSCIG 20% with unwarmed 1.0 g/kg IgG 20%** | | | |
| --- | --- | --- | --- | --- | --- | --- | --- | --- | --- | --- | --- | --- |
|  | **BMI 18–<25 kg/m^2^ (*n* = 3)** | | **BMI 25–30 kg/m^2^ (*n* = 5)** | | **BMI 18–<25 kg/m^2^ (*n* = 5)** | | **BMI 25–30 kg/m^2^ (*n* = 3)** | | **BMI 18–<25 kg/m^2^ (*n* = 4)** | | **BMI 25–30 kg/m^2^ (*n* = 4)** | |
|  | ***n*** | **Value** | ***n*** | **Value** | ***n*** | **Value** | ***n*** | **Value** | ***n*** | **Value** | ***n*** | **Value** |
| **Actual total IgG 20% dose,^a^ mean (SD), g** | 3 | 26.7 (2.1) | 5 | 29.2 (2.7) | 5 | 58.8 (5.9) | 3 | 83.3 (8.5) | 4 | 63.3 (11.0) | 4 | 78.8 (13.8) |
| **Total IgG 20% volume infused,^a^ mean (SD), mL** | 3 | 133.3 (10.4) | 5 | 146.0 (13.4) | 5 | 294.0 (29.5) | 3 | 416.7 (42.5) | 4 | 316.3 (55.1) | 4 | 393.8 (69.0) |
| Infusion site 1 | 3 | 133.3 (10.4) | 5 | 146.0 (13.4) | 5 | 286.0 (16.4) | 3 | 300.0 (0.0) | 4 | 287.5 (25.0) | 4 | 300.0 (0.0) |
| Infusion site 2^b^ | 0 | NA | 0 | 0 (NA) | 1 | 40.0 (NA) | 3 | 116.7 (42.5) | 3 | 38.3 (40.4) | 4 | 93.8 (69.0) |
| **Total rHuPH20 volume infused,^a^ mean (SD), mL** | 3 | 13.7 (1.2) | 5 | 14.6 (1.3) | 5 | 29.6 (2.9) | 3 | 42.0 (4.00) | 4 | 32.0 (5.7) | 4 | 39.5 (7.1) |
| Infusion site 1 | 3 | 13.7 (1.2) | 5 | 14.6 (1.3) | 5 | 28.8 (1.6) | 3 | 30.0 (0.0) | 4 | 28.8 (2.5) | 4 | 30.0 (0.0) |
| Infusion site 2^b^ | 0 | NA | 0 | NA | 1 | 4.0 (NA) | 3 | 12.0 (4.0) | 3 | 4.3 (4.0) | 4 | 9.5 (7.1) |
| **Total infusion duration,^a^ mean (SD), minutes** | 3 | 61.3 (3.2) | 5 | 67.2 (5.2) | 5 | 104.2 (17.5) | 3 | 159.3 (11.1) | 4 | 126.0 (26.1) | 4 | 152.5 (22.2) |
| Infusion site 1 | 3 | 61.3 (3.2) | 5 | 67.2 (5.2) | 5 | 97.2 (3.3) | 3 | 101.3 (2.1) | 4 | 102.0 (8.6) | 4 | 152.5 (22.2) |
| Infusion site 2^b^ | 0 | N/A | 0 | N/A | 1 | 35.0 (NA) | 3 | 58.0 (10.5) | 3 | 32.0 (16.5) | 4 | 50.0 (21.4) |

^a^Combined data for infusion sites 1 and 2. ^b^A total of 0, 4 and 7 patients used a second infusion site in treatment arms 1, 2 and 3, respectively.

Abbreviations: *BMI*, body mass index; *fSCIG 20%*, facilitated immunoglobulin 20%; *IgG*, immunoglobulin G; *N/A*, not applicable; *rHuPH20*, recombinant human hyaluronidase; *SD*, standard deviation
